# Toxicological responses of A549 and HCE-T cells exposed to fine particulate matter at the air–liquid interface

**DOI:** 10.1007/s11356-024-32944-4

**Published:** 2024-03-21

**Authors:** Wankang Chen, Pengxiang Ge, Minjun Deng, Xiaoming Liu, Zhenyu Lu, Zhansheng Yan, Mindong Chen, Junfeng Wang

**Affiliations:** 1 Jiangsu Key Laboratory of Atmospheric Environment Monitoring and Pollution Control, Collaborative Innovation Center of Atmospheric Environment and Equipment Technology, School of Environmental Science and Engineering, Nanjing University of Information Science & Technology, Nanjing, 210044 China; 2 Ningxia Meteorological Service Center, Yinchuan, 750002 China

**Keywords:** PM_2.5_, Air–liquid interface, Human epithelial cells, Apoptosis, Inflammatory damage, DNA damage

## Abstract

Fine particulate matter (PM_2.5_) can enter the human body in various ways and have adverse effects on human health. Human lungs and eyes are exposed to the air for a long time and are the first to be exposed to PM_2.5_. The “liquid immersion exposure method” has some limitations that prevent it from fully reflecting the toxic effects of particulate matter on the human body. In this study, the collected PM_2.5_ samples were chemically analyzed. An air–liquid interface (ALI) model with a high correlation to the in vivo environment was established based on human lung epithelial cells (A549) and immortalized human corneal epithelial cells (HCE-T). The VITROCELL Cloud 12 system was used to distribute PM_2.5_ on the cells evenly. After exposure for 6 h and 24 h, cell viability, apoptosis rate, reactive oxygen species (ROS) level, expression of inflammatory factors, and deoxyribonucleic acid (DNA) damage were measured. The results demonstrated significant dose- and time-dependent effects of PM_2.5_ on cell viability, cell apoptosis, ROS generation, and DNA damage at the ALI, while the inflammatory factors showed dose-dependent effects only. It should be noted that even short exposure to low doses of PM_2.5_ can cause cell DNA double-strand breaks and increased expression of γ-H2AX, indicating significant genotoxicity of PM_2.5_. Increased abundance of ROS in cells plays a crucial role in the cytotoxicity induced by PM_2.5_ exposure These findings emphasize the significant cellular damage and genotoxicity that may result from short-term exposure to low levels of PM_2.5_.

**Supplementary Information:**

The online version contains supplementary material available at 10.1007/s11356-024-32944-4.

## Introduction

With urbanization and industrialization on the rise, environmental pollution, particularly air pollution, has escalated worldwide. This pressing issue has garnered increasing attention due to its detrimental impact on the human body. In 2013, the International Agency for Research on Cancer (IARC) listed atmospheric particulates as the first category of carcinogens. Fine particulate matter (PM_2.5_) has a smaller size, a larger specific surface area, and a greater ability to adsorb harmful organic matter and heavy metals, which is more detrimental to human health compared to inhalable particles (PM_10_) (Deng et al. 2013; Deng et al. 2014). China is one of the countries most affected by PM_2.5_, with 1.27 million premature deaths due to PM_2.5_ pollution each year (Pang et al. 2020; Wang et al. 2017). Epidemiological studies have provided evidence that once PM_2.5_ enters the human body, it can reach the deep alveoli in the lungs and penetrate the alveolar area. Subsequently, these particles may potentially be absorbed by the bloodstream and disseminated throughout the body, thereby contributing to the development of various respiratory and cardiovascular diseases in humans (Bengalli et al. 2013). It can also penetrate the blood–brain barrier and placental barrier, causing harm to the human nervous system and reproductive system (Wang et al. 2022). Furthermore, recent research has brought to light the detrimental effects of PM_2.5_ on the surface of the eyes, thereby contributing to the emergence of various eye conditions (Yang et al. 2019). Studies have shown that PM_2.5_ can deposit on the eyelids and eyelashes (Muruganandam et al. 2023). Short-term or long-term exposure to an environment with excessive air quality can cause eye irritation (eye itching, tears, etc.) and lead to diseases such as dry eye disease, conjunctivitis, and pterygium. Numerous studies have shown that the health effects caused by PM_2.5_ are closely related to its composition. PM_2.5_ is composed of a complex blend of water-soluble ions, carbonaceous compounds, organic substances, heavy metals, and other elements. Among these, water-soluble ions are a significant constituent of PM_2.5_ and have the potential to impact cell viability, lactic dehydrogenase levels (LDH), and reactive oxygen species (ROS) (Zou et al. 2016). Heavy metals, such as chromium (Cr), arsenic (As), lead (Pb), and cadmium (Cd), have been found to have detrimental effects on human health due to their carcinogenic and teratogenic properties. Additionally, they can accumulate within the body, leading to harm to the reproductive system (Mukherjee et al. 2022; Sui et al. 2020; Zhang et al. 2016). Polycyclic aromatic hydrocarbons (PAHs) can lead to oxidative stress, inflammatory reactions, and deoxyribonucleic acid (DNA) damage in cells. Studies have indicated that short-term exposure to high concentrations of PAHs can lead to diarrhea and eye discomfort, and long-term exposure can cause damage to the human immune system and lungs (Abdel-Shafy and Mansour 2016).

Due to the substantial time and cost associated with conventional in vivo inhalation and animal experiments, there is a heightened interest in utilizing in vitro methods to evaluate the toxicity of PM_2.5_. Currently, most of the in vitro models for evaluating PM_2.5_ toxicity are the “liquid immersion exposure method,” which directly uses the medium to expose PM_2.5_ to cells to assess the cytotoxicity of PM_2.5_. However, it is important to note that this method has certain limitations. For example, it demonstrates poor repeatability and may result in changes in particle size due to the accumulation of PM_2.5_, thereby potentially inadequately reflecting the toxicity of particulate matter to the human body (Bihari et al. 2008). Brownian motion of particles in the medium prevents particles from settling on cells completely, which may underestimate the cytotoxicity of particles (Muehlfeld et al. 2008). Another method of cell exposure is the air–liquid interface (ALI) method, which allows cells to grow at the interface between air and liquid through the use of a Transwell chamber. Compared with traditional in vitro exposure, ALI exposure can avoid the problems encountered in traditional exposure experiments. It can make direct contact between particles and cells, which is more in line with the real state of cells in the body (Guenette et al. 2022). Our previous studies have also demonstrated that PM_2.5_ has better dispersion and deposition properties at the ALI model (Chen et al. 2024). Studies have shown that when cells are cultured at the ALI, they develop highly differentiated functions and structures (Aufderheide 2005). These findings underscore the significance of investigating the toxic effects of PM_2.5_ on both the respiratory and ocular systems, employing advanced research methods like the ALI exposure model.

In this study, a comprehensive analysis was conducted on the chemical composition of the collected PM_2.5_ samples. To simulate exposure scenarios in real life, human lung epithelial cells (A549) and immortalized human corneal epithelial cells (HCE-T) were exposed to different doses of PM_2.5_ at the ALI. Following exposures of 6 and 24 h, several key cytotoxic indicators were assessed, including cell viability, ROS levels, cell apoptosis rate, expression of inflammatory factors, and DNA damage. The primary objective of our research was to evaluate the dose- and time-response relationships concerning the cellular toxicity of PM_2.5_ on both the respiratory and ocular systems at the ALI. It is crucial for assessing the potential risks associated with PM_2.5_ exposure and improving the understanding of its impact on the respiratory and ocular systems.

## Materials and methods

### Collection of particulate matter

The PM_2.5_ samples were collected at Nanjing University of Information Science & Technology (N 32° 15′ 11″, E 118° 42′ 49″), and a total of 80 samples were collected from December 2021 to November 2022. The samples were collected on quartz microfiber filters (Whatman, UK). The collection process involved the use of a large-flow sampler (Jinshida, Qingdao, China), which was set to operate at a flow rate of 1050 L/min. To prepare the quartz microfiber filters for use, they underwent a process of baking in a muffle furnace at a temperature of 450 ℃ for a duration of 8 h. The purpose of this procedure was to effectively remove any organic matter present on the filters. The filters were weighed both before and after sampling and were put into a desiccator for 24 h of drying and balancing before weighing. The accuracy of the balance used for weighing is one in 100,000, and the filters should be weighed three times with an error not exceeding 0.05 mg. The collected quartz microfiber filters were cut into pieces, and ultrasonic extraction was carried out in ultrapure water for 20 min and repeated 3 times. After ultrasound treatment, the suspension was filtered using 8 layers of sterile gauze. The filtered solution was then transferred into a freeze-drying machine and subjected to a 1-week process to obtain the PM_2.5_ samples. The samples were sealed in centrifugal tubes and stored in a − 20 ℃ refrigerator away from light for later use.

### Composition analysis of PM_2.5_

The PM_2.5_ samples were placed in the centrifuge tubes, sonicated with ultrapure water, and then filtered through a 0.22 μm filter. The content of water-soluble ions in the samples was determined by an ion chromatograph (ICS-3000, ICS-2000, Dionex, Sunnyvale, CA, USA). The organic carbon (OC) and elemental carbon (EC) in PM_2.5_ samples were determined using a carbon analyzer (Sunset Model 4, Sunset Laboratory Inc., Forest Grove, OR, USA). After 65% HNO_3_ was added to the particles for microwave digestion, an inductively coupled plasma mass spectrometry was used to determine the concentration of heavy metals in samples (ICP-MS, Agilent Technologies, USA). The content of PAHs in samples was determined by a gas chromatography-mass spectrometer (GC–MS, Agilent Technologies, USA). PM_2.5_ samples added with CH_2_Cl_2_ and internal standard solution were sonicated, filtered, and rotary evaporated. 200 μL of CH_2_Cl_2_ was then added to fix the volume, and a total of 16 PAHs were detected in the samples. The standard curves of all measured substances displayed excellent linearity (*r*^2^ > 0.999). The recovery rates of all the measured substances fell within the acceptable range of 100 ± 15%.

### Cell culture

A549 cells were obtained from Stem Cell Bank, Chinese Academy of Sciences. A549 cells were cultured in RPMI-1640 medium (Bio-Channel, China), which was supplemented with 10% fetal bovine serum (FBS, BIOAGRIO SCIENCE, China). HCE-T cells were obtained from Wuhan Punosai Life Technology Co., LTD. HCE-T cells were cultured in the DMEM/F12 medium (Bio-Channel, China), which was supplemented with 10% FBS, 5 μg/mL Insulin (Beyotime, China), and 10 ng/mL human epidermal growth factor (EGF, BIOAGRIO SCIENCE, China). The cell culture was maintained in an incubator (Thermo Scientific, MA, USA) at a constant temperature of 37 ℃ and 5% CO_2_. The supplemented RPMI-1640 and DMEM/F12 medium were further cited as complete cell medium (CCM).

A549 and HCE-T cells were passaged when 80–90% confluent. For all the exposure experiments, 50,000 cells/insert were cultured in 12-well culture plate inserts. These inserts have a surface area of 1.12 cm^2^ and are equipped with a 0.4 μm pore polyester (PET) membrane (LABSELECT, China). For each well, 0.5 mL of CCM was added to the upper side and 1.5 mL to the basal side for each well. The cells were incubated for 24 h under immersion conditions, and the medium in the upper side was then removed after forming a tight contact cell layer. The cells were incubated for12 h at the ALI until the exposure experiment was conducted.

### Cell exposure

The exposure experiments were performed in the VITROCELL Cloud 12 system (VITROCELL SYSTEMS, Waldkirch, Germany); detailed information of which is available on the vendor’s website (www.Vitrocell.com). 4 mL of the basal medium was added to each exposed module of the VITROCELL Cloud 12 system so that the liquid level was consistent with the height of the PET membrane to ensure sufficient contact between medium and cells. The exposure dose of PM_2.5_ was determined by the cell growth area of the insert, and the deposition mass of PM_2.5_ on the insert was 25, 50, and 100 μg in this study. The Transwell chamber was put into the exposure module, and the prepared PM_2.5_ solution was quickly and evenly exposed to the cells using the atomizer (Aeroneb Lab; Aerogen, Galway, Ireland). Complete exposure could be achieved within 5 min. The Cloud 12 system has electric heating, which can maintain 37 ℃ throughout the exposure process to provide a favorable environment for cells to survive. After ALI exposure was complete, the CCM in the basal side was replaced with 1 mL basal medium and placed in an incubator for 6 h or 24 h until cytotoxic indicators were determined.

### Detection of cell viability

Cell Counting Kit-8 kit (CCK-8, Beyotime, China) was used to measure cell viability. After exposure, 1 mL of the mixture CCK-8 and the medium (CCK-8 concentration of 10%) was added to each upper chamber and incubated at 37 °C for a duration of 2 h. After the incubation, the mixtures from each chamber were transferred to separate wells in a 96-well plate. The optical density of the samples was measured at a specific wavelength of 450 nm using a Microplate Reader (ID3, SpectraMax, USA), and the results were normalized.

### Detection of apoptosis rate

Apoptosis was determined using Annexin V-FITC apoptosis assay kit (Beyotime, China). After exposure, the cells were washed with PBS once, digested with EDTA-free trypsin, and collected into a centrifuge tube. Centrifuge at 1000 r/min for 5 min, resuspend and count 100,000 cells, and centrifuge again. 195 μL of Annexin V-FITC binding solution, 5 μL of Annex V-FITC, and 10 μL of propidium iodide (PI) staining solution were added to suspend the cells. Double-negative group, Annex V-FITC single-staining group, and PI single-staining group were set up for localization of cells. The double-negative group used cells that were not exposed to PM_2.5_, and the single-stained group used cells from the exposed group. The cells were gently mixed and assayed by flow cytometry (Beckman, CA, USA) at 10,000 cells per assay.

### Detection of ROS

To detect ROS levels, the DCFH-DA probe (Sigma, MO, USA) was utilized. DCFH-DA was diluted in the basal medium to a final concentration of 10 μM. In each well, 500 μL of the diluted DCFH-DA solution was added, and the cells were then incubated at 37 ℃ for a duration of 20 min. To ensure the removal of excess DCFH-DA, the cells were washed 2 times with phosphate buffer saline (PBS). Then, digest the cells with trypsin without EDTA, suspend with 500 μL PBS, and transfer to a flow tube. Fluorescence intensity was measured by flow cytometry, with excitation wavelength at 488 nm and emission wavelength at 525 nm. 5000 cells were measured each time and the results were normalized.

### Detection of inflammatory factor

TNF-α and IL-6 are inflammatory factors that play a significant role in particulate matter-induced inflammatory damage. TNF-α is a pre-inflammatory response factor that can promote the secretion of inflammatory factor IL-6, and the overexpression of IL-6 is related to the inflammation of cells (Möller and Villiger 2006). The inflammatory factor levels in cell culture medium were determined using an enzyme-linked immunosorbent assay (ELISA) kit (Jiangsu Meimian, China) to judge the immunosorbent damage effects of particulate matter on cells. The optical density at 450 nm was measured using a Microplate Reader, and the results were normalized.

### Detection of DNA damage

H2AX is a variant of the histone protein H2A. H2AX phosphorylates when a cell’s DNA double strand breaks. The level of γ-H2AX produced by H2AX phosphorylation reflects the degree of DNA damage. γ-H2AX staining showed green fluorescence. DNA damage was determined by γ-H2AX immunofluorescence assay, using the DNA damage detection kit (Beyotime, China). Finally, images were captured under an inverted fluorescence microscope (NE900, Jiangnan Yong Xin Corporation, China). Fluorescence intensity was quantified using ImageJ (National Institutes of Health, MD, USA) to calculate the mean pixel intensity of nuclear area, ultimately resulting in normalization.

### Statistical analysis

In this study, each experiment was repeated a minimum of 3 times, and the experimental data were expressed as mean ± standard deviation (SD). Statistical analysis was performed by using SPSS 26.0 (SPSS Inc., USA). The differences in cytotoxicity between different concentration groups and time groups were analyzed by one-way analysis of variance (ANOVA). *p* < 0.05 implies statistically significant and *p* < 0.01 was extremely significant.

## Results

### Mass concentration analysis of different chemical components in PM_2.5_

**Fig. 1:**
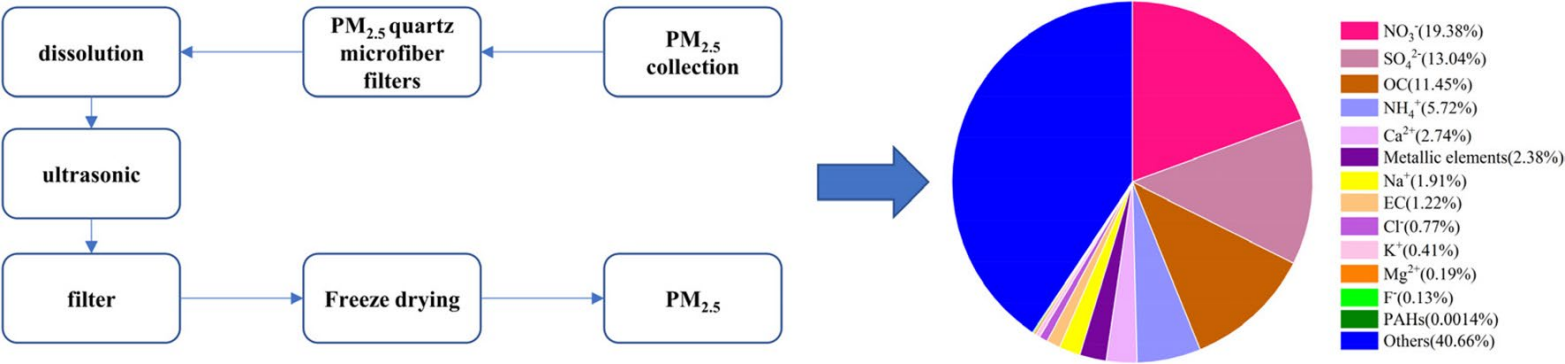
Chemical composition of PM_2.5_

The average mass concentration of PM_2.5_ collected was 36.96 μg/m^3^, and the main components of PM_2.5_ are shown in Fig. 1. Water-soluble ions accounted for 44.29% of the total mass of PM_2.5_, of which SO_4_^2−^, NO_3_^−^, and NH_4_^+^ (SNA) particles account for the highest proportion. Atmospheric carbon comes in the form of OC and EC, both of which are important components of PM_2.5_. OC and EC account for 11.45% and 1.22% of the total mass of PM_2.5_. The OC/EC ratio is commonly employed to determine the secondary source contribution to PM_2.5_. The OC/EC ratio of PM_2.5_ was 9.39, indicating that the PM_2.5_ in Jiangbei New Area of Nanjing was dominated by the secondary generation. A total of 32 kinds of metallic elements were determined in PM_2.5_, accounted for 2.38% of the mass of PM_2.5_. The content of 16 kinds of PAHs in PM_2.5_ was also determined, and the PAH content accounted for 0.01‰ of the mass of PM_2.5_. Although PAHs make up only a small fraction of PM_2.5_, they are harmful to the human body, causing damage to the respiratory and nervous systems. For more detailed information on water-soluble ions, OCEC, metallic elements, and PAHs, please refer to the Online Resource 1, 2, 3, and 4.

### PM_2.5_ decreases epithelial cell viability

**Fig. 2:**
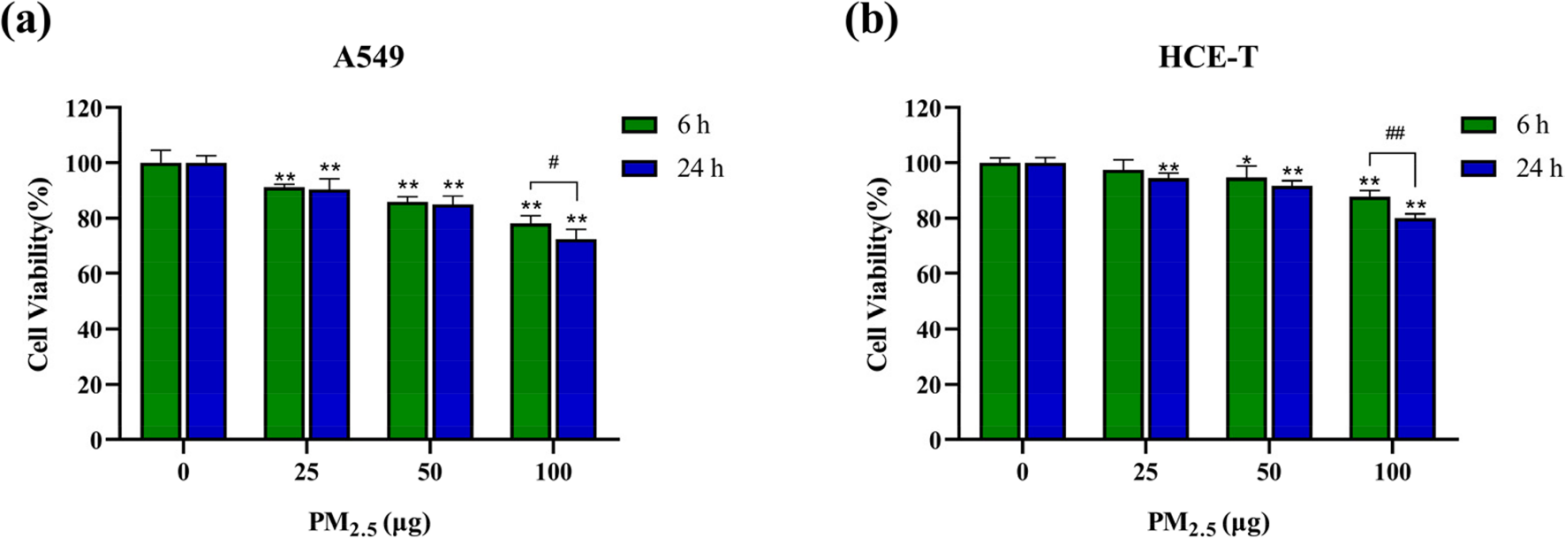
Cell viability after exposure to PM_2.5_ for 6 h and 24 h (*n* = 3). **a** A549 cells. **b** HCE-T cells. * and # means *p* < 0.05, ** and ## means *p* < 0.01

As depicted in Fig. 2, for A549 cells, exposure to PM_2.5_ for 6 h and 24 h resulted in a significant decrease in cell viability in each dose group compared to the control group. For HCE-T cells, only the 25 μg dose group exposed for 6 h showed no significant decrease in cell viability, while the cell viability in all other dose groups significantly decreased. We observed that as the dose of PM_2.5_ increased, the inhibitory effect on cell viability became more pronounced, indicating the harmful impact of PM_2.5_ on cells. Furthermore, at the same exposure time and dose, the cell viability of A549 cells was lower than that of HCE-T cells, with the greatest difference observed at a dose of 100 μg and an exposure time of 6 h, where A549 cell viability was 9.6% lower than that of HCE-T cells, suggesting higher sensitivity of A549 cells to PM_2.5_ stimulation. With increasing exposure time, A549 and HCE-T cells showed significant differences in cell viability at a dose of 100 μg, while the differences between other exposure groups were not statistically significant. These findings indicate that PM_2.5_ exhibits greater cytotoxicity on A549 cells compared to HCE-T cells. Exposure to medium and low doses of PM_2.5_ for 6 h resulted in a decrease in cell viability similar to that caused by exposure for 24 h. However, as the exposure dose increased, longer exposure to PM_2.5_ had a greater impact on cell viability.

### PM_2.5_ causes apoptosis in epithelial cells

**Fig. 3:**
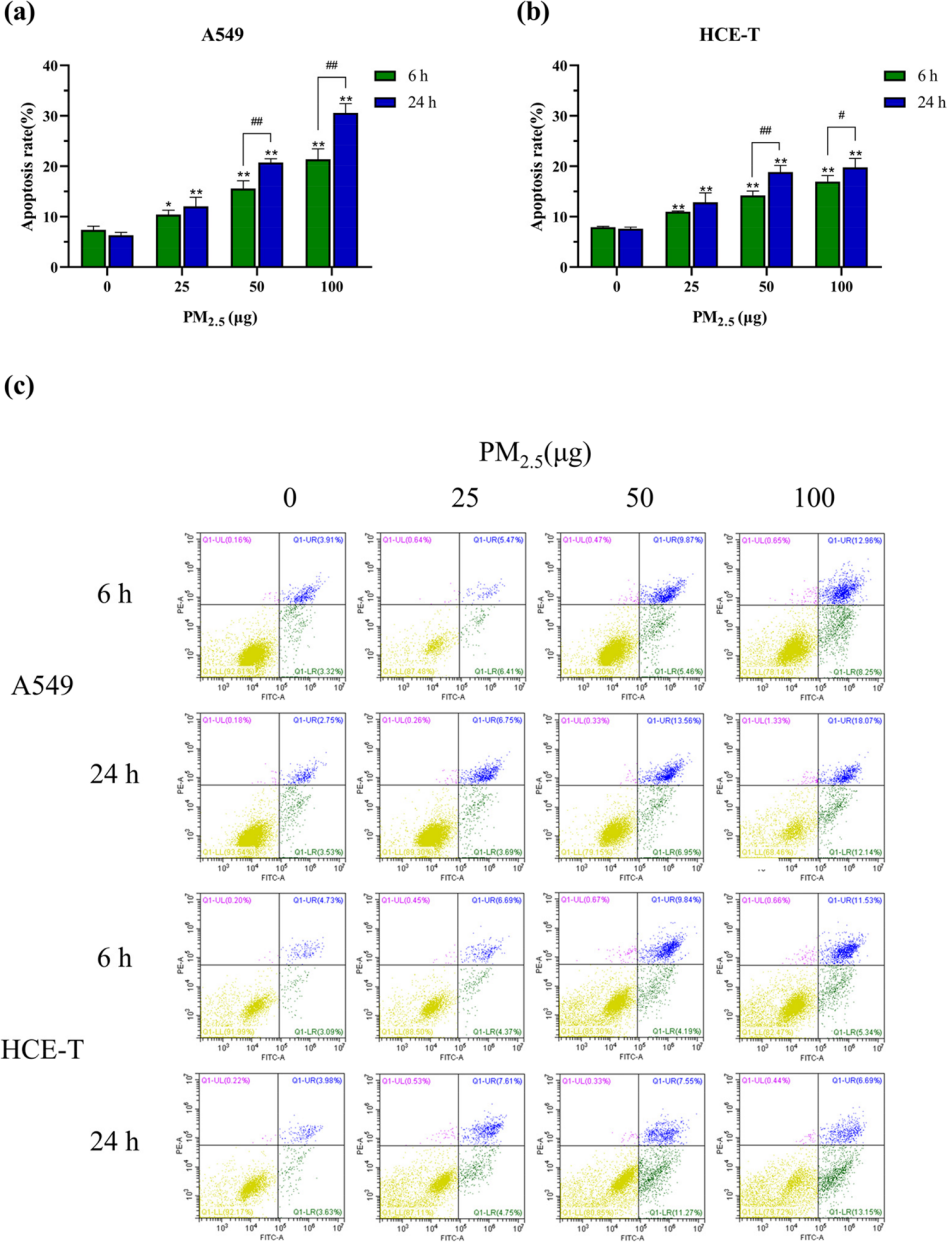
Apoptosis rates after exposure to PM_2.5_ for 6 h and 24 h (*n* = 3). **a** A549 cells. **b** HCE-T cells. **c** Flow cytogram of apoptosis rates. * and # means *p* < 0.05, ** and ## means *p* < 0.01

Exposure to PM_2.5_ typically results in cellular apoptosis and necrosis. The data in Fig. 3 show that the apoptosis rates of both cell types showed a significant dose dependence as the PM_2.5_ exposure dose increased. In the medium and high-dose exposure groups, the apoptosis rate of cells increased significantly with prolonged exposure duration. In addition, the apoptosis rates of A549 and HCE-T cells were similar at medium and low-dose exposures. When the dose was increased to 100 μg, the difference in apoptosis rates between the two cells gradually increased. The apoptosis rate of A549 cells was higher than that of HCE-T by 4.43% at an exposure time of 6 h. When the exposure time was increased to 24 h, the maximum difference in apoptosis rates of the two cells appeared, with 30.59% for A549 cells and 19.79% for HCE-T cells, which was 10.8% different from each other. The results indicated that the apoptosis rate of A549 cells was higher with the increase of exposure dose.

### PM_2.5_ promotes ROS generation in epithelial cells

**Fig. 4:**
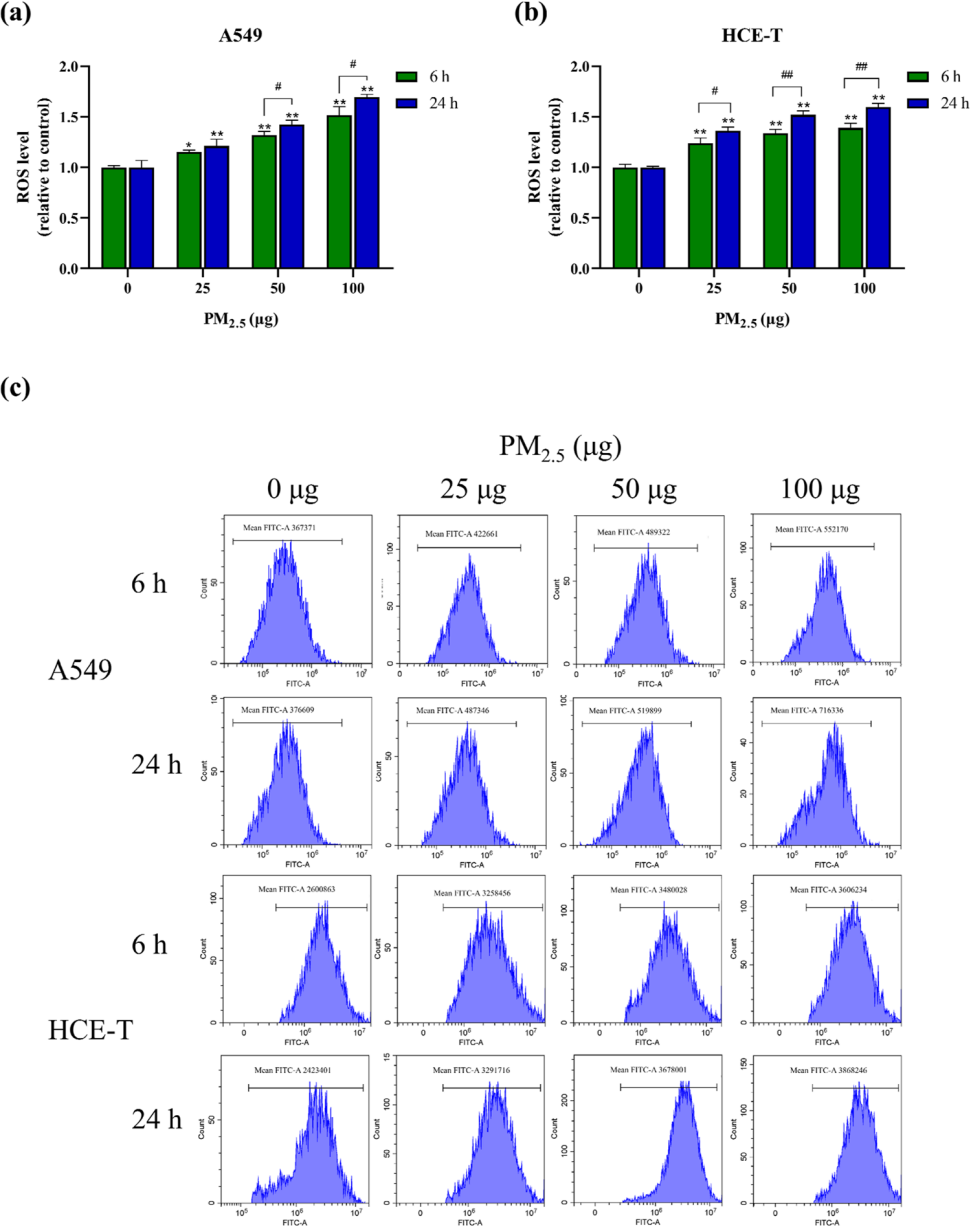
ROS generation level after exposure to PM_2.5_ for 6 h and 24 h (*n* = 3). **a** A549 cells. **b** HCE-T cells. **c** Flow cytogram of ROS. * and # means *p* < 0.05, ** and ## means *p* < 0.01

Figure 4 shows the level of ROS generation. After exposure to PM_2.5_, ROS levels showed a significant dose-dependent increase in A549 and HCE-T cells compared to the control. Whether exposed for 6 h or 24 h, HCE-T cells exhibited higher levels of ROS at low doses of PM_2.5_ exposure, whereas A549 cells exhibited higher levels of ROS as the exposure dose increased. These findings suggest that HCE-T cells are more sensitive to ROS at low doses of PM_2.5_ exposure, while A549 cells experience more pronounced oxidative damage at high doses. Interestingly, the trend in ROS levels parallels that of apoptosis rates, with the increase of exposure time, the ROS level of cells significantly increases, suggesting that the cells were subjected to more severe oxidative damage with increasing exposure time.

### PM_2.5_ induces inflammatory factor expression in epithelial cells

**Fig. 5:**
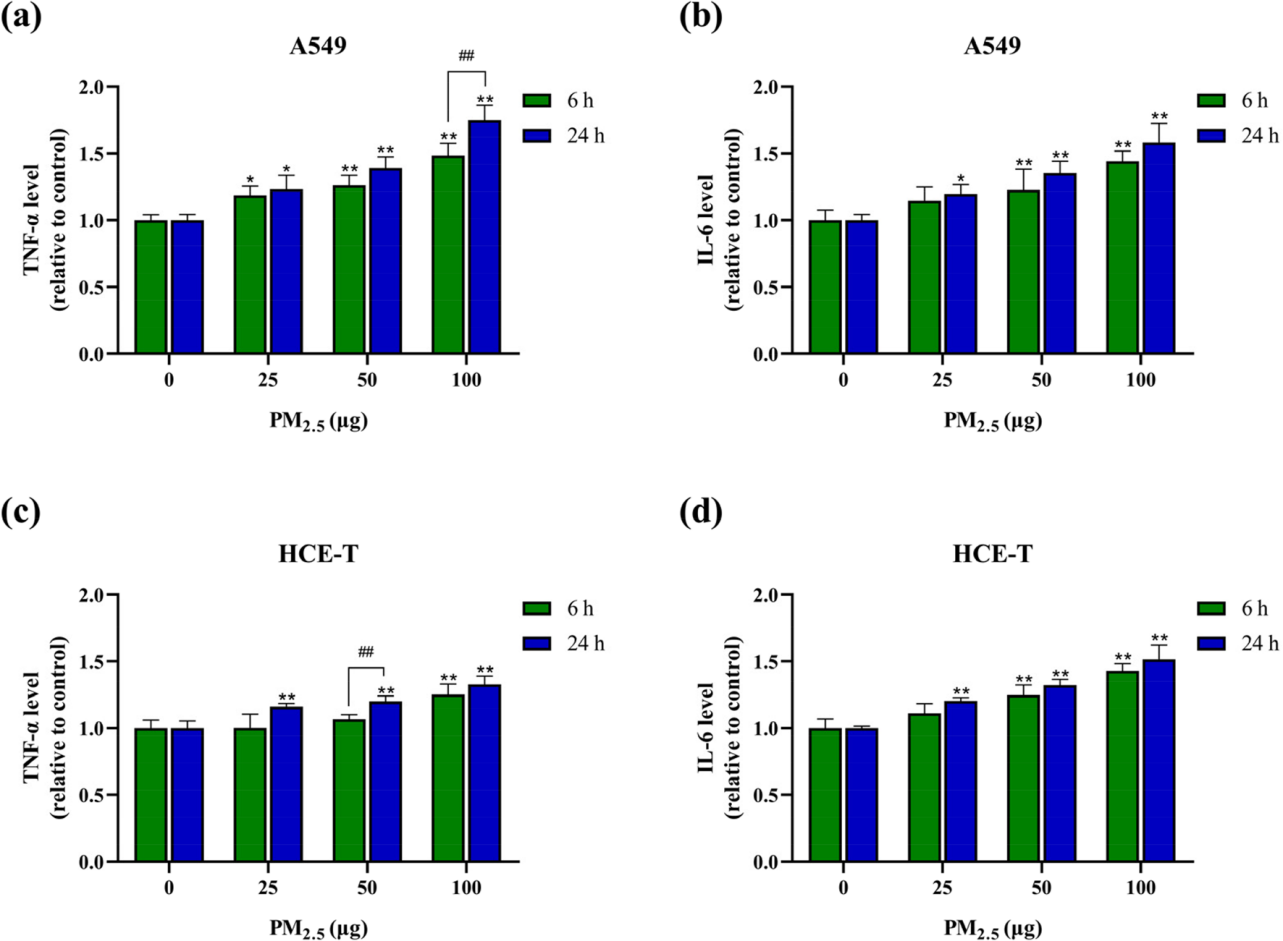
Expression levels of inflammatory factors after exposure to PM_2.5_ for 6 h and 24 h (*n* = 3). **a** TNF-α level in A549 cells. **b** IL-6 level in A549 cells. **c** TNF-α level in HCE-T cells. **d** IL-6 level in HCE-T cells. * and # means *p* < 0.05, ** and ## means *p* < 0.01

Current studies have generally shown that the expression level of inflammatory factors increases after oxidative stress occurs in cells (Lugrin et al. 2014). To this end, we determined the expression levels of TNF-α and IL-6 in the cell culture medium. As shown in Fig. 5, for A549 cells, TNF-α level increased significantly with increasing exposure dose after 6 and 24 h of exposure to PM_2.5_. IL-6 level showed no significant difference only after 6 h of low-dose exposure, and the other exposure groups showed significant difference compared with the control group. For HCE-T cells, after 6 h of exposure, TNF-α level was significantly increased only when exposed to a dose of 100 μg, whereas IL-6 level began to increase significantly at an exposure of 50 μg. After 24 h of exposure, TNF-α and IL-6 levels in HCE-T cells of all dose exposure group were statistically different from those of the control group. The results indicated that the expression levels of TNF-α and IL-6 of the two cells were significantly dose-dependent. Notably, there was no particularly significant difference in the expression of inflammatory factors in the two types of cells with the increase in exposure time, suggesting that inflammation-induced cellular damage occurs already after 6 h of exposure.

### PM_2.5_ causes DNA damage in epithelial cells

**Fig. 6:**
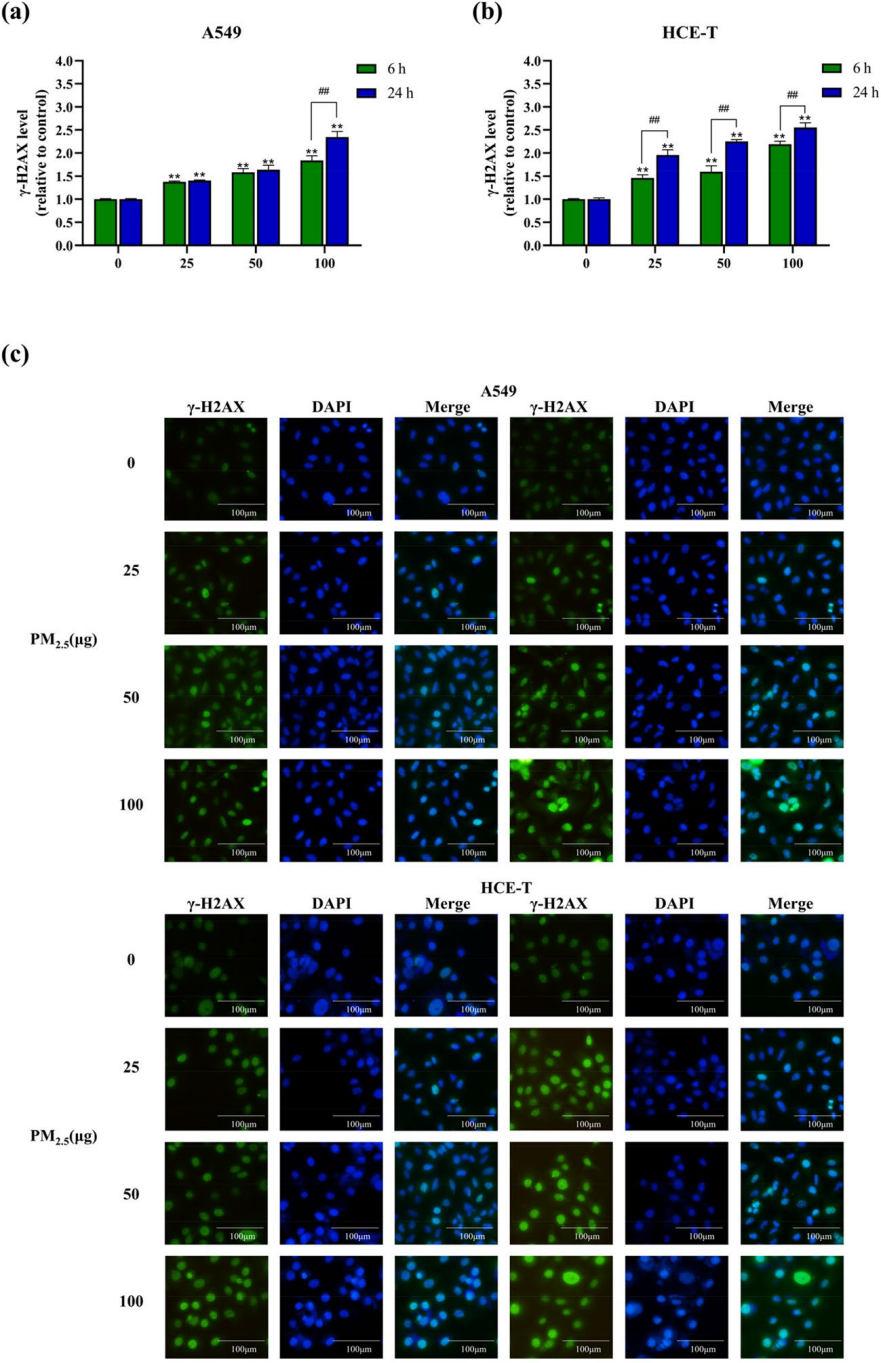
Levels of cellular γ-H2AX production after exposure to PM_2.5_ for 6 h and 24 h (*n* = 3). **a** A549 cells. **b** HCE-T cells. **c** Cellular γ-H2AX fluorescence intensity after exposure to PM_2.5_. * and # means *p* < 0.05, ** and ## means *p* < 0.01

In addition, this study also measured the damage of PM_2.5_ to cellular DNA. DNA double-strand breaks are considered to be the most severe DNA damage. When cellular DNA is damaged, H2AX is phosphorylated to produce γ-H2AX, which fluoresces green when stained. The fluorescence intensity can be calculated to reflect the degree of DNA damage. As shown in Fig. 6, the levels of γ-H2AX increased dose-dependently in both cells at different exposure times. For A549 cells, only the 100 μg dose group showed a significant increase in γ-H2AX levels with increasing exposure time, and no significant differences were observed in the low and medium-dose groups. For HCE-T cells, the extent of γ-H2AX levels after 24 h of exposure to PM_2.5_ was significantly higher than that after 6 h of exposure. The results indicated that a low dose of PM_2.5_ could lead to DNA damage in HCE-T cells compared with A549 cells and that HCE-T cells had a more pronounced time dependence of PM_2.5_-induced DNA damage.

## Discussion

Previous studies have shown that the ALI model has good applicability in in vitro toxicology, and compared to traditional immersion exposure, the ALI model can more accurately simulate the real environment of the human body. Currently, the ALI method has been used in numerous studies to investigate the effects of air pollution and particulate matter mixtures on lung cell models (Gonzalez-Rivera et al. 2020; Kaur et al. 2022; Leclercq et al. 2016; Offer et al. 2022; Tomasek et al. 2016). For the human body, long-term exposure of the lungs and eyes system to the atmosphere can easily affect various pollutants in the atmosphere, leading to a series of health risks. Human epithelial cells such as A549 and HCE-T cells serve as the first barrier of the human body, playing a role as a barrier and sentinel. Currently, extensive and profound research has been conducted on the cytotoxic effects of PM_2.5_ on A549 and HCE-T cells under immersion exposure. However, epithelial cells were exposed to air for a long period of time, and using traditional immersion exposure to evaluate the cytotoxicity of PM_2.5_ clearly does not meet the requirements. Current research has shown that in traditional immersion exposure, the duration of exposure significantly affects the cytotoxicity of particulate matter, leading to decreased cell activity and increased inflammatory response (Rahmatinia et al. 2022). However, at the ALI, the debate is not unanimous. Loret and Wang et al. pointed out that exposure at the ALI for 3 h is sufficient to observe adverse cellular reactions, including cytotoxicity, oxidative stress, and inflammatory damage, after 24 h of exposure (Loret et al. 2016; Wang et al. 2019). However, Miao et al. found that HCE cells exposed to cigarette extracts exhibited time-dependent cytotoxicity under ALI (Miao et al. 2019). Therefore, exposure time is still crucial for studying the cytotoxicity of PM_2.5_ at the ALI. Therefore, in this study, a three-dimensional model of human epithelial cells was created using A549 and HCE-T cells, exposing the cells to PM_2.5_ during ALI. The effects of PM_2.5_ on human epithelial cell activity, cell apoptosis rate, ROS levels, inflammatory factor expression levels, and DNA damage were measured.

The cell viability results showed that after exposure to PM_2.5_, the cell viability of A549 and HCE-T cells decreased in a dose- and time-dependent manner. The dose-dependent decrease in cell viability is consistent with other studies, with a dose-dependent decrease in 16HBE cell viability after exposure to PM_2.5_ for 24 h (Niu et al. 2020). The results of cell apoptosis showed that with the increase of PM_2.5_ exposure dose and exposure time, the apoptosis rates of both types of cells significantly increased, indicating that PM_2.5_ has a time- and dose-dependent effect on cell apoptosis. It can be seen that A549 cells exhibited lower cell viability and higher apoptosis rates with increasing PM_2.5_ exposure dose compared to HCE-T cells. A recent study showed that the apoptosis rate induced by PM_2.5_ is higher than that of PM_10_. Shan et al. exposed cells to PM_2.5_ after treatment with antioxidants (NAC, N-acetylcysteine) and found that NAC significantly reduced the apoptosis rate after PM_2.5_ exposure, indicating that PM_2.5_-induced apoptosis is related to the generation of ROS (Shan et al. 2021). Another study suggests that ROS can induce cell apoptosis by participating in signaling pathways such as P53 and Caspase. Liu et al.’s research suggests that oxidative stress can trigger ATM/P53/CDK2 and mitochondrial apoptosis pathways to induce apoptosis in rat spermatocytes (Liu et al. 2019). Qi et al. believe that PM_2.5_ can induce apoptosis and injury in A549 cells by activating the MAPK/NF-xB/STAT1 pathway (Qi et al. 2019).

Oxidative stress is considered one of the key mechanisms of cell damage, and the possible cause of cell apoptosis is mitochondrial stress induced by oxidative stress, which in turn leads to cell apoptosis (Liu et al. 2019). ROS is a product of normal cellular metabolism. When cells are stimulated and produce excessive ROS, this balance is disrupted, resulting in oxidative damage to the cells. Previous reports have shown that excessive ROS generation plays an important role in PM_2.5_-induced cytotoxicity (Abbas et al. 2019). To further investigate the cytotoxic mechanism of PM_2.5_ at the ALI, we measured the ROS generation levels of two types of cells after PM_2.5_ exposure. Our research results indicate that PM_2.5_ increases ROS generation, and ROS generation is dose- and time-dependent. HCE-T cells were more sensitive at low doses, and A549 cells exhibited higher ROS levels as the exposed dose increased. As the exposure time increases, the ROS levels of HCE-T cells show greater temporal differences. This is consistent with our previous research findings that an increase in PM_2.5_ concentration can induce ROS generation in mouse spermatocytes (Ge et al. 2023). Other studies using different cell lines have also shown that exposure to PM_2.5_ can cause an increase in cell ROS and inflammatory damage (Jia et al. 2023; Jin et al. 2018; Kim et al. 2022; Qiu et al. 2019). It should be noted that previous studies have shown that A549 cells are one of the most heterologous cells in many lung cell models compared to lung tissue and primary lung epithelial cells. Therefore, there may be significant differences in ROS generation and accumulation between A549 cells and in vivo lung tissue (Courcot et al. 2012). In our previous research, we found that water-soluble ions such as SO_4_^2−^, NO_3_^−^, and NH_4_^+^ showed a strong positive correlation with ROS (Di et al. 2020). In the PM_2.5_ used in this study, SO_4_^2−^, NO_3_^−^, and NH_4_^+^ accounted for 13.04%, 16.45%, and 5.72%, respectively. They are the main components of water-soluble ions. They are relatively more likely to enter cells, leading to oxidative stress. The carbon components present in PM_2.5_, such as elemental carbon and organic carbon, can also have cytotoxic effects on cells. Elemental carbon nanoparticles have been shown to induce oxidative stress in cells and trigger an inflammatory response, leading to cell dysfunction, DNA damage, and apoptosis. Recent studies have emphasized the important contribution of metals in oxidative stress, such as nickel (Ni), copper (Cu), Pb, Cd, and As (Crobeddu et al. 2020; Fang et al. 2021). Metals will adsorb on PM_2.5_ and induce ROS generation when inhaled into the lungs or deposited on the surface of the eyes. Al and Pb have been proven to be related to redox reactions, while zinc (Zn) can inhibit lead-induced oxidative stress (Bondy and Kirstein 1996; Zhang 2023). Some carcinogenic metals, such as Cr and Cd, induce ROS generation, which in turn leads to DNA damage (Lin et al. 2007; Wakeman 2006; Wang et al. 1997). Composite heavy metal exposure can lead to more severe oxidative stress than single heavy metal exposure (Sun et al. 2015). In addition, PAHs have been shown to induce ROS generation in cells (He et al. 2022; Onduka et al. 2017). Although PAHs have a low proportion in PM_2.5_, they have high toxicity and carcinogenicity. Previous studies have shown that PAHs with high ring numbers have higher toxicity.

In addition to oxidative stress, inflammatory damage is also considered an important mechanism of cell damage and a major precursor to cell apoptosis (Yin et al. 2015). Therefore, we measured TNF-α and IL-6 levels in the supernatant of A549 cells and HCE-T cells. The results showed a significant dose-dependent relationship between TNF-α and IL-6 levels in both types of cells, and A549 cells are more sensitive to the expression of inflammatory factors than HCE-T cells. Meanwhile, A549 cells are more sensitive to the increase in cell apoptosis rate after PM_2.5_ exposure. According to reports, metals in particulate matter are the main components that induce inflammatory damage, and the release of inflammatory factors is related to the content of Cu and Zn (Deng et al. 2023; Hosseini et al. 2021; Schneider et al. 2020). The water-soluble components in PM_2.5_ can quickly enter cells and cause cellular inflammatory damage. NH_4_^+^ can disrupt cell homeostasis by altering intracellular pH, induce apoptosis, activate cellular inflammatory responses leading to cell damage (Lagadic-Gossmann et al. 2004; Shrode et al. 1997). PM_2.5_ can trigger endothelial cell activation by upregulating the IL-6 dependent JAK1/STAT3 pathway (Hu et al. 2016). Inflammatory damage can also be induced by activating transcription factors such as NF-kB and AP-1 (Potnis et al. 2013; Xu et al. 2019). These studies indicate that exposure to PM_2.5_ can induce cellular inflammatory damage and subsequently induce cell apoptosis. Some studies have shown that oxidative damage caused by high exposure to PM_2.5_ may be a key factor in the high expression of inflammatory factors. ROS is an important messenger that activates various cellular signaling pathways and can activate the expression of inflammatory genes. In addition, PM_2.5_-induced inflammatory damage can be significantly inhibited by antioxidants, indicating that the release of cytokines requires oxidative stress (Santos et al. 2015). Inconsistent research results demonstrate that the complex components of PM_2.5_ are important participants in PM_2.5_-induced inflammatory damage.

Due to the presence of carcinogenic metals and PAHs in PM_2.5_, studying the genotoxicity of PM_2.5_ is particularly important. These metals can induce DNA damage and impair DNA repair mechanisms, leading to genomic instability and increased risk of disease (Cavallo et al. 2009). A recent study has shown that γ-H2AX levels appear to increase with increasing cadmium levels in PM samples (Engels et al. 2023). PAHs are highly toxic and carcinogenic (Chen et al. 2021; Yu et al. 2015). PAHs can penetrate cells and produce reactive metabolites that can covalently bind to DNA, resulting in genotoxicity (Bai et al. 2017; Stading et al. 2021). This DNA damage can lead to mutations and lead to the development of cancer. When DNA in a cell is damaged, a phosphorylation event occurs on a histone variant called H2AX, resulting in its transformation into γ-H2AX. The expression level of γ-H2AX can directly reflect the degree of DNA damage and repair. DNA damage can be assessed by detecting γ-H2AX. In this study, we used immunofluorescence to detect DNA damage and found that γ-H2AX expression was significantly increased in A549 and HCE-T cells exposed to PM_2.5_, suggesting that A549 and HCE-T cells exhibited significant DNA damage at low doses of acute exposure, which became increasingly severe with increasing exposure time and dose. In addition, the DNA-damaging effect of PM2.5 on HCE-T cells showed a greater time dependence than that of A549 cells. Yang et al. found that PM_2.5_ in six Chinese cities induced γ-H2AX production in human bronchial epithelioid cells (BEAS-2B) (Yang et al. 2016). Borgie et al. collected PM_2.5_ from rural and urban Lebanon and found that both showed induction of γ-H2AX, and PM_2.5_ showed significant genotoxicity to cells (Borgie et al. 2015). This also supports our results. ROS-mediated oxidative damage is considered to be one of the important mechanisms of DNA damage in cells. In our research results, we measured ROS and found that PM_2.5_ induced ROS generation, disrupting the homeostasis of cellular oxidative stress. We observed that HCE-T cells exhibited significant time-dependent effects on the γ-H2AX level compared to A549 cells. This observation is consistent with the generation of ROS. It suggests that HCE-T cells have a higher sensitivity to ROS-induced DNA damage, as reflected by the increased γ-H2AX level over time. Recent studies have also shown that PM_2.5_ induces DNA double-strand breaks in HCEC and 16HBE cells and increases the expression of repair protein γ-H2AX through ROS accumulation and oxidative stress. Gao et al. found that PM_2.5_ can cause significant DNA damage in HCECs, and ROS inhibitor NAC can partially eliminate this effect, indicating that PM_2.5_-induced oxidative stress plays an important role in DNA damage (Gao et al. 2016).

## Conclusion

This study showed that PM_2.5_ induced cytotoxic effects on A549 and HCE-T cells through cell viability, apoptosis rate, oxidative stress, inflammation damage, and genotoxicity at the ALI. The results indicate that PM_2.5_ has dose-and time-dependent effects on cell viability, apoptosis rate, ROS generation, and DNA damage, while inflammatory factors only exhibit dose-dependent effects. A549 cells were more sensitive to apoptosis and inflammation, while HCE-T cells exhibited rapid expression of ROS and γ-H2AX. Increased abundance of ROS in cells is crucial in PM_2.5_-induced cytotoxicity. Further research is needed to assess the potential risks to human health of each component of PM_2.5_ and the exact mechanisms involved and develop effective strategies to mitigate the health risks posed by PM_2.5_ on humans.

### Supplementary Information

Below is the link to the electronic supplementary material.

Supplementary file1 (DOCX 14 KB)

Supplementary file2 (DOCX 14 KB)

Supplementary file3 (DOCX 15 KB)

Supplementary file4 (DOCX 15 KB)

## Data Availability

Available upon request by contacting the author.
