# Supplementary material for: Toxicological responses of A549 and HCE-T cells exposed to fine particulate matter at the air–liquid interface
Source: Environ Sci Pollut Res Int. 2024 Mar 21;31(18):27375–87. doi: 10.1007/s11356-024-32944-4 (PMC11052810; doi:10.1007/s11356-024-32944-4)
Supplement: Supplementary file 1 — Supplementary file1 (DOCX 14 KB) [file 11356_2024_32944_MOESM1_ESM.docx]

**Table S1** Mass concentration of Water-soluble ions in PM_2.5_

| Water-soluble ions | Mass concentration (mg/g) | Water-soluble ions | Mass concentration (mg/g) |
| --- | --- | --- | --- |
| Na^+^ | 19.0955 | F^-^ | 1.3146 |
| NH_4_^+^ | 57.1782 | Cl^-^ | 7.6895 |
| K^+^ | 4.1151 | SO_4_^2-^ | 130.3618 |
| Mg^2+^ | 1.8616 | NO_3_^-^ | 193.7956 |
| Ca^2+^ | 27.4384 |  |  |
