# Supplementary material for: Toxicological responses of A549 and HCE-T cells exposed to fine particulate matter at the air–liquid interface
Source: Environ Sci Pollut Res Int. 2024 Mar 21;31(18):27375–87. doi: 10.1007/s11356-024-32944-4 (PMC11052810; doi:10.1007/s11356-024-32944-4)
Supplement: Supplementary file 2 — Supplementary file2 (DOCX 14 KB) [file 11356_2024_32944_MOESM2_ESM.docx]

**Table S2** Mass concentration of Carbon contents in PM_2.5_

| Carbon contents | Mass concentration (mg/g) |
| --- | --- |
| OC | 114.49 |
| EC | 12.25 |
