# Supplementary material for: Toxicological responses of A549 and HCE-T cells exposed to fine particulate matter at the air–liquid interface
Source: Environ Sci Pollut Res Int. 2024 Mar 21;31(18):27375–87. doi: 10.1007/s11356-024-32944-4 (PMC11052810; doi:10.1007/s11356-024-32944-4)
Supplement: Supplementary file 3 — Supplementary file3 (DOCX 15 KB) [file 11356_2024_32944_MOESM3_ESM.docx]

**Table S3** Mass concentration of PAHs in PM_2.5_

| PAHs | Mass concentration (μg/g) | PAHs | Mass concentration (μg/g) |
| --- | --- | --- | --- |
| Naphthalene | 0.271344 | Benzo[a]anthracene | 0.679095 |
| Acenaphthene | 0.448785 | Chrysene | 1.258038 |
| Acenaphthylene | 0.193526 | Benzo[b&k]fluoranthene | 1.311053 |
| Fluorene | 0.513854 | Benzo[a]pyrene | 0.794736 |
| Phenanthrene | 0.879657 | Indeno[1,2,3-cd]pyrene | 1.068817 |
| Anthracene | 0.332843 | Benzo[g,h,i]perylene | 1.544741 |
| Fluoranthene | 1.311273 | Dibenzo[a,h]anthracene | 0.402252 |
| Pyrene | 2.813266 |  |  |
