# Supplementary material for: Toxicological responses of A549 and HCE-T cells exposed to fine particulate matter at the air–liquid interface
Source: Environ Sci Pollut Res Int. 2024 Mar 21;31(18):27375–87. doi: 10.1007/s11356-024-32944-4 (PMC11052810; doi:10.1007/s11356-024-32944-4)
Supplement: Supplementary file 4 — Supplementary file4 (DOCX 15 KB) [file 11356_2024_32944_MOESM4_ESM.docx]

**Table S4** Mass concentration of Metallic elements in PM_2.5_

| Metallic elements | Mass concentration (mg/g) | Metallic elements | Mass concentration (mg/g) |
| --- | --- | --- | --- |
| Fe | 11.68546 | V | 0.041097 |
| Al | 5.89699 | Sb | 0.023878 |
| Zn | 2.867908 | W | 0.015995 |
| B | 0.781684 | Bi | 0.011786 |
| Mn | 0.745714 | Cd | 0.010867 |
| Pb | 0.233801 | Ce | 0.009031 |
| Cu | 0.216276 | Co | 0.006429 |
| Ti | 0.190561 | La | 0.004821 |
| Li | 0.185663 | Tl | 0.004362 |
| Ba | 0.168673 | Nd | 0.003673 |
| Sr | 0.16102 | Sn | 0.003597 |
| Cr | 0.139133 | Ga | 0.003291 |
| As | 0.090918 | Y | 0.002985 |
| Mo | 0.089847 | Cs | 0.002449 |
| Ni | 0.080357 | Nb | 0.001607 |
| Se | 0.071556 | Pr | 0.000842 |
